# Supplementary material for: T cell responses at diagnosis of amyotrophic lateral sclerosis predict disease progression
Source: Nat Commun. 2022 Nov 8;13:6733. doi: 10.1038/s41467-022-34526-9 (PMC9643478; doi:10.1038/s41467-022-34526-9)
Supplement: Supplementary file 1 — Supplementary Information [file 41467_2022_34526_MOESM1_ESM.pdf]

Supplementary Information

T cell responses at diagnosis of amyotrophic lateral sclerosis predict disease progression

Solmaz Yazdani[1†], Christina Seitz[1†], Can Cui[1†], Anikó Lovik[1], Lu Pan[2], Fredrik Piehl[3,4], Yudi Pawitan[2], Ulf Kläppe[3,4], Rayomand Press[3,4], Kristin Samuelsson[3,4], Li Yin[2], Trung Nghia Vu[2], Anne-Laure Joly[3], Lisa S. Westerberg[5], Björn Evertsson[3,4], Caroline Ingre[3,4†], John Andersson[1†*], Fang Fang[1†*]

1) Institute of Environmental Medicine, Karolinska Institutet, Stockholm, Sweden
2) Department of Medical Epidemiology and Biostatistics, Karolinska Institutet, Stockholm, Sweden
3) Department of Clinical Neuroscience, Karolinska Institutet, Stockholm, Sweden
4) Neurology clinic, Karolinska University Hospital, Stockholm, Sweden
5) Department of Microbiology, Tumor, and Cell Biology, Karolinska Institutet, Stockholm, Sweden

† Equally Contributing Authors

[*] Corresponding authors:

John Andersson or Fang Fang

Unit of Integrative Epidemiology

Institute of Environmental Medicine, Karolinska Institutet

Box 210, 171 77 Stockholm, Sweden

Phone: +46 8 5248 6131

Email: john.andersson@ki.se or fang.fang@ki.se

Supplementary Figure 1

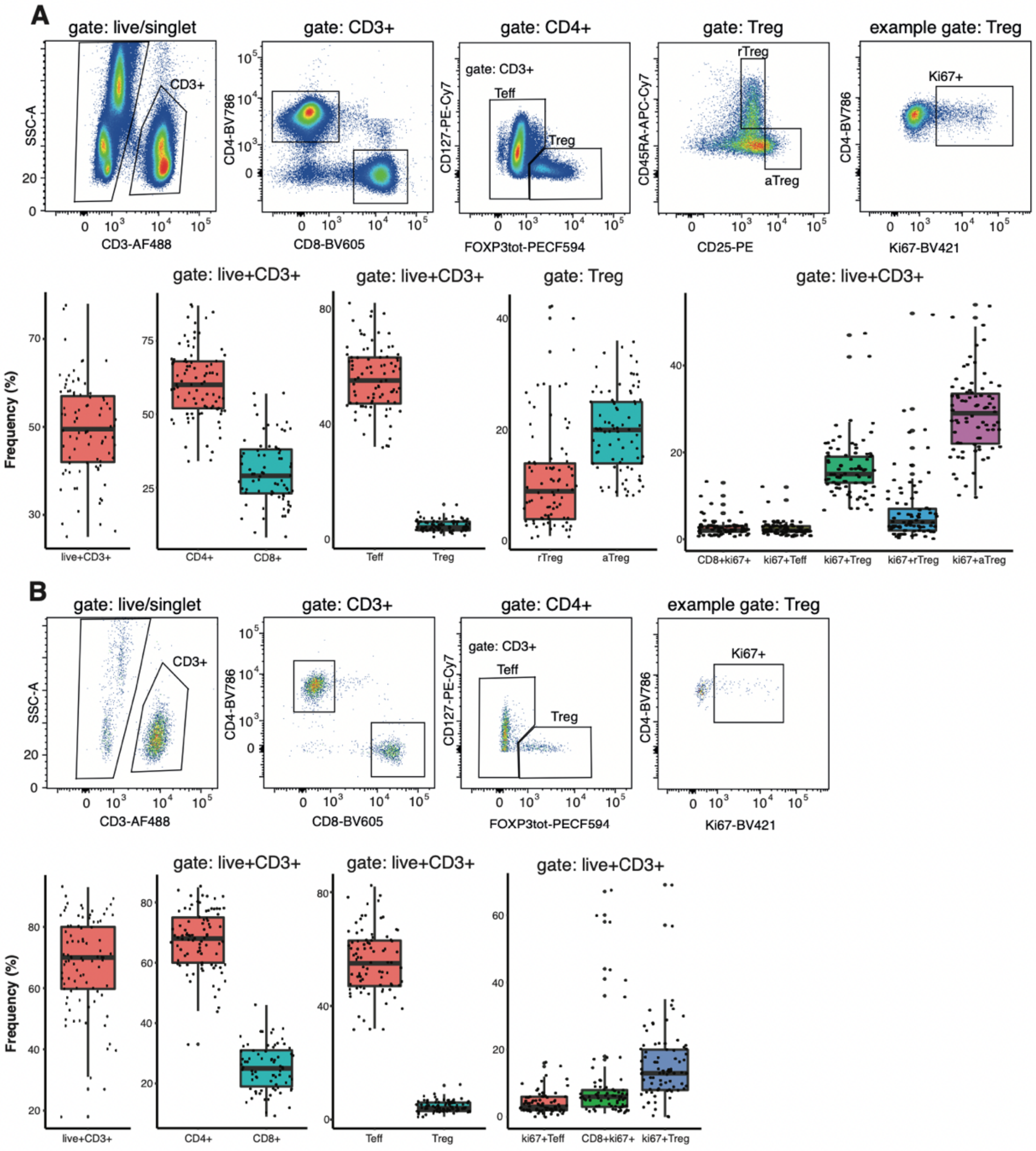

C

Cell population out of live cells (%)	Blood		CSF	
	Number of patients	Mean (SD)	Number of patients	Mean (SD)
CD3+	86	49.60 (10.79)	88	68.07 (15.15)
CD4+	86	29.97 (8.10)	88	46.39 (14.02)
CD8+	72	14.61 (6.51)	73	16.63 (5.74)
Teff	84	27.50 (7.46)	86	43.73 (13.14)
Treg	84	2.31 (1.00)	86	46.39 (14.02)
aTreg	83	0.45 (0.22)		
rTreg	84	0.27 (0.25)		
aTreg/rTreg (ratio)	83	3.72 (4.06)		

Figure S1. Gating strategy and summary of primary data for T cell subsets. Flow cytometry was used to define distinct T cell subsets. Gating strategy and distribution of T cell subsets in blood (A) and cerebrospinal fluid (B). Mean and standard deviation (SD) of frequencies of T cell subsets (C). The lower, midline and upper hinges of the boxplots are representative of 25th, 50th and 75th percentiles, respectively. The lower and upper whiskers extend to the lowest and largest values but no more than 1.5 * inter-quartile range. The box plots represent the 25th, 50th and 75th percentiles with N= the number of patients for each variable listed in table C.

Supplementary Figure 2

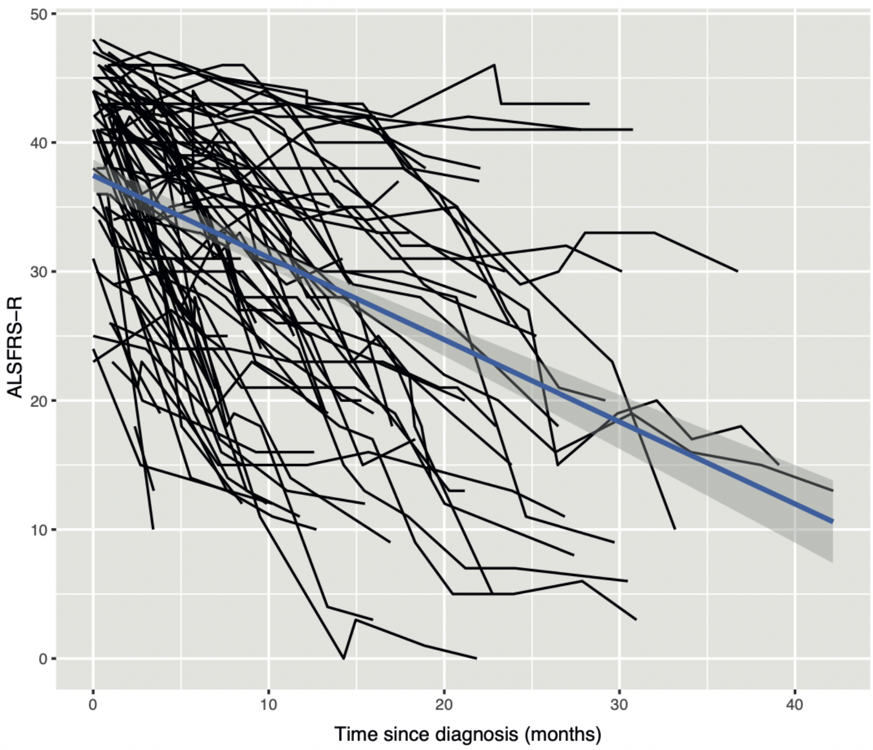

Figure S2. ALSFRS-R score declines over time. ALS functional rating scale-revised (ALSFRS-R) score was plotted against time from diagnosis for all the 89 patients included in the study. Mean evolution and 95% confidence interval is shown as the continuous line and the shaded area.

Supplementary Figure 3

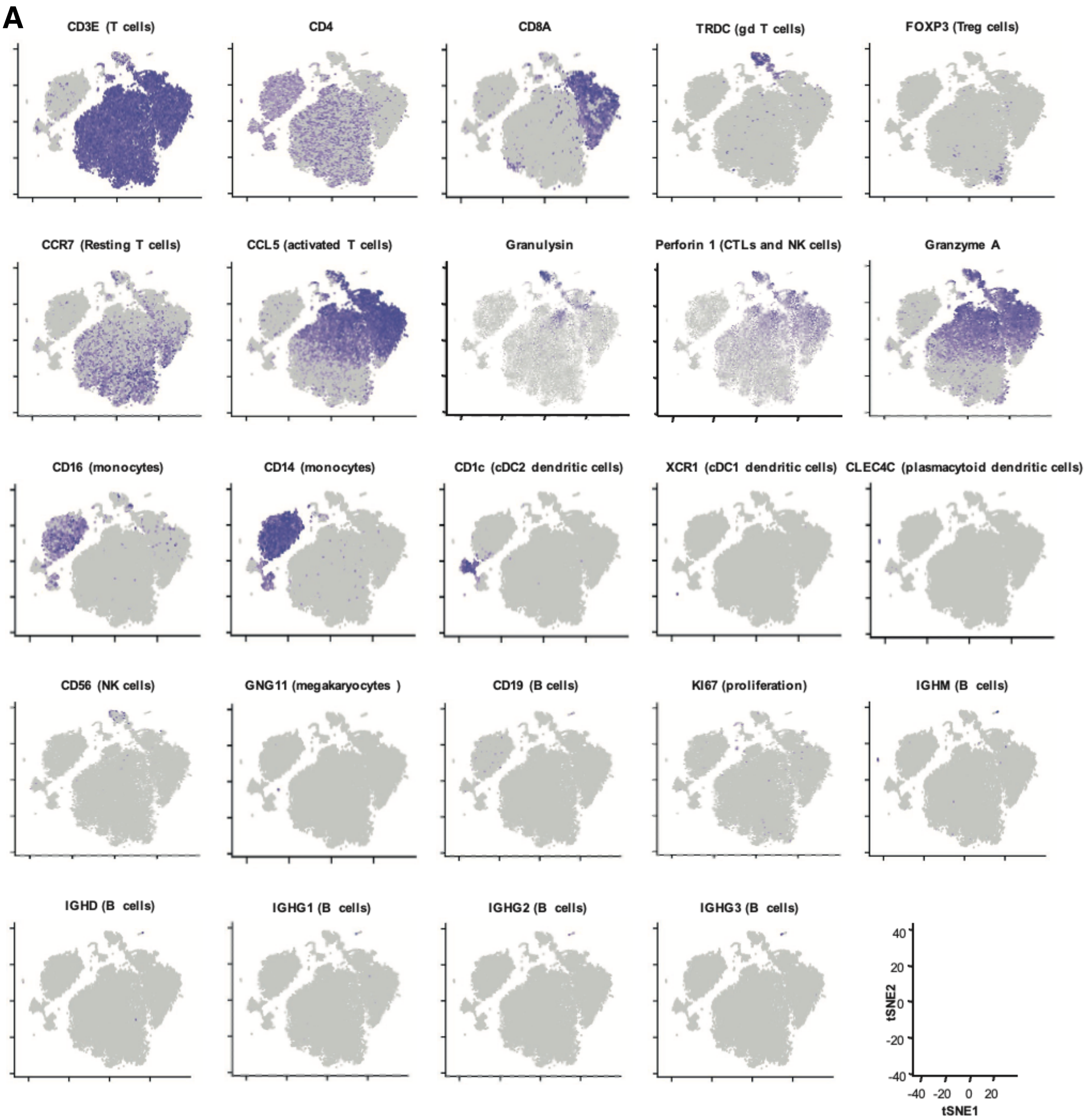

B

Cell type	Gene	Cell type	Gene
B cells	CD19, IGHM, IGHD, IGHG1-3	Proliferating T cells	CD3E MKI67 (KI67)
Megakaryocyte	GNG11	Regulatory T cells	CD3E, CD4, FOXP3
CD16+ monocytes	CD14, FCGR3A (CD16)+	gd T cells	CD3E, TRDC
CD16- monocytes	CD14, FCGR3A (CD16)-	CD4 CTL	CD3E, CD4, Perforin
cDC1 dendritic cells	XCR1	DP T cells	CD3E, CD4, CD8,
cDC2 dendritic cells	CD1c	Activated CD4+ T cells	CD3E, CD4, CCR7-, CCL5+
pDC	CLEC4C	Non-activated CD4+ T cells	CD3E, CD4, CCR7+, CCL5-
CD16+ dendritic cells	CD14, CD16	Activated CD8+ T cells	CD3E, CD8, CCR7-
NK cells	NCAM1 (CD56), Perforin	Non-activated CD8+ T cells	CD3E, CD8, CCR7+

Figure S3. Gene expression for manual cell type annotation. t-SNE plots of 10X scRNAseq data from all patients combined (A), showing expression genes used for the manual annotation of cell populations (B).

Supplementary Table 1. Hazard ratios (HRs) and their 95% confidence intervals (CIs) of death in relation to per standard deviation increase in the frequency of T cell subsets in blood or cerebrospinal fluid (CSF), after adjustment for age at diagnosis, sex, site of onset, delay in diagnosis, disease progression rate at diagnosis, body mass index (BMI), and time difference between measurement of BMI and blood sampling.

Cell population out of live cells (%)	Blood		CSF	
	No. of patients (deaths)	HR (95% CI)	No. of patients (deaths)	HR (95% CI)
$CD3^+$	78 (44)	1.36 (0.93-1.98)	80 (46)	1.17 (0.86-1.59)
$CD4^+$	78 (44)	**1.49 (1.05-2.11)**	80 (46)	1.25 (0.91-1.74)
$CD8^+$	66 (35)	0.96 (0.65-1.43)	67 (37)	0.98 (0.70-1.39)
$CD4^+$/$CD8^+$ (ratio)	67 (36)	1.35 (0.93-1.96)	67 (37)	1.18 (0.77-1.80)
T_{eff}	77 (44)	**1.58 (1.10-2.27)**	79 (46)	1.29 (0.93-1.78)
Treg	77 (44)	1.03 (0.79-1.33)	79 (46)	0.86 (0.57-1.30)
aTreg	76 (44)	0.94 (0.70-1.27)		
rTreg	77 (44)	1.24 (0.93-1.66)		
aTreg/rTreg (ratio)	76 (44)	**0.63 (0.41-0.98)**		

Supplementary Table 2. Hazard ratios (HRs) and their 95% confidence intervals (CIs) of death in relation to per standard deviation increase in the frequency of T cell subsets in blood or cerebrospinal fluid, adjusted for age at diagnosis, sex, site of onset, delay in diagnosis, disease progression rate at diagnosis, body mass index (BMI), and time difference between measurement of BMI and blood sampling, after excluding ALS mimics.

Cell population out of live cells (%)	Blood		CSF	
	No. of patients (deaths)	HR (95% CI)	No. of patients (deaths)	HR (95% CI)
$CD3^+$	73 (44)	**1.54 (1.04-2.26)**	75 (46)	1.27 (0.93-1.75)
$CD4^+$	73 (44)	**1.65 (1.15-2.36)**	75 (46)	1.36 (0.98-1.89)
$CD8^+$	62 (35)	0.93 (0.62-1.39)	63 (37)	1.04 (0.74-1.46)
$CD4^+/CD8^+$ (ratio)	63 (36)	1.41 (0.98-2.01)	63 (37)	1.17 (0.77-1.78)
T_{eff}	72 (44)	**1.70 (1.17-2.46)**	74 (46)	1.37 (0.99-1.88)
Treg	72 (44)	1.13 (0.85-1.50)	74 (46)	0.99 (0.65-1.49)
aTreg	71 (44)	1.14 (0.81-1.59)		
rTreg	72 (44)	1.26 (0.93-1.70)		
aTreg/rTreg (ratio)	71 (44)	0.67 (0.44-1.02)		

Supplementary Table 3. Hazard ratios (HRs) and their 95% confidence intervals (CIs) of death in relation to per standard deviation increase in the frequency of T cell subsets in blood or cerebrospinal fluid, adjusted for age at diagnosis, sex, site of onset, delay in diagnosis, disease progression rate at diagnosis, body mass index (BMI), and time difference between measurement of BMI and blood sampling, after excluding patients with known *C9orf72* mutation.

Cell population out of	Blood		CSF	
live cells (%)	Individuals	HR (95% CI)	Individuals	HR (95% CI)
$CD3^+$	68 (40)	1.35 (0.91-2.00)	70 (42)	1.08 (0.80-1.47)
$CD4^+$	68 (40)	**1.49 (1.04-2.12)**	70 (42)	1.13 (0.80-1.59)
$CD8^+$	56 (31)	0.93 (0.62-1.41)	57 (33)	1.02 (0.70-1.50)
$CD4^+$/$CD8^+$ (ratio)	57 (32)	1.38 (0.93-2.04)	57 (33)	0.99 (0.61-1.63)
T_{eff}	68 (40)	**1.57 (1.08-2.29)**	70 (42)	1.16 (0.82-1.62)
Treg	68 (40)	1.04 (0.80-1.36)	70 (42)	0.85 (0.55-1.31)
aTreg	67 (40)	0.94 (0.69-1.29)		
rTreg	68 (40)	1.28 (0.92-1.79)		
aTreg/rTreg (ratio)	67 (40)	0.62 (0.38-1.01)		

Supplementary Table 4. Characteristics of the entire study cohort and the cohort including only patients with no missing data, according to the Swedish Motor Neuron Disease Quality Registry.

**Characteristics**	**Entire study cohort** (n=89)	**After exclusion of patients with missing data** (n=63)
**Age at diagnosis, years**		
Mean (SD)	66.52 (10.69)	66.32 (11.68)
**Sex, N (%)**		
Male	54 (60.67%)	39 (61.90%)
Female	35 (39.33%)	24 (38.10%)
**Final diagnosis, N (%)**		
ALS	82 (92.13%)	59 (93.65%)
Other MND	7 (7.87%)	4 (6.35%)
**Site of onset, N (%)**		
Bulbar	38 (42.70%)	28 (44.44%)
Non-bulbar	51 (57.30%)	35 (55.56%)
Other	-	
**ALSFRS-R score at diagnosis, mean (SD)**	38.29 (7.85)	38.20 (8.11))
**Progression rate at diagnosis, mean (SD)**	0.81 (0.82)	0.80 (0.77)
**Diagnostic delay, median**	377 days	376 days
**Survival status at end of follow-up, N (%)**		
Dead	50 (56.18%)	33 (52.38%)
Alive	39 (43.82%)	30 (47.62%)

Supplementary Table 5. Hazard ratios (HRs) and their 95% confidence intervals (CIs) of death (or use of invasive ventilation) in relation to frequency of T cell subsets in blood or cerebrospinal fluid (CSF), after adjustment for age at diagnosis, sex, site of onset, diagnostic delay, disease progression rate at diagnosis, body mass index (BMI), and time difference between measurement of BMI and blood sampling, analysis using the entire study cohort and after exclusion of patients with missing data.

**Cell population out of live cells (%)**	**Tertile**	**Entire study cohort (n=89)**		**After exclusion of patients with missing data (n=63)**	
		No. of patients (outcomes)	HR (95% CI)	No. of patients (outcomes)	HR (95% CI)
			**Blood**		
CD3+	Low	26 (12)	Ref	21 (11)	Ref
	Medium	26 (16)	**2.47 (1.11-5.52)**	21 (12)	2.41 (0.92-6.36)
	High	26 (16)	**2.49 (1.03-6.03)**	21 (12)	1.84 (0.70-4.81)
CD4+	Low	26 (13)	Ref	21 (10)	Ref
	Medium	26 (15)	1.82 (0.81-4.06)	21 (15)	**2.92 (1.25-6.79)**
	High	25 (16)	**2.29 (1.04-5.04)**	21 (10)	1.71 (0.66-4.41)
CD8+	Low	22 (13)	Ref	21 (13)	Ref
	Medium	22 (13)	1.04 (0.44-2.46)	21 (13)	1.11 (0.48-2.57)
	High	22 (09)	0.81 (0.32-2.06)	21 (9)	0.74 (0.29-1.86)
Teff	Low	26 (13)	Ref	21 (11)	Ref
	Medium	26 (14)	1.61 (0.72-3.62)	21 (13)	2.06 (0.88-4.83)
	High	25 (17)	**2.43 (1.10-5.37)**	21 (11)	1.79 (0.72-4.47)
Treg	Low	26 (12)	Ref	21 (10)	Ref
	Medium	26 (14)	1.44 (0.64-3.23)	21 (11)	1.65 (0.67-4.10)
	High	25 (18)	1.39 (0.64-3.00)	21 (14)	1.28 (0.55-2.97)
aTreg	Low	26 (15)	Ref	21 (11)	Ref
	Medium	25 (12)	**0.40 (0.17-0.92)**	21 (09)	0.55 (0.22-1.42)
	High	25 (17)	0.76 (0.37-1.59)	21 (15)	1.03 (0.45-2.35)
rTreg	Low	26 (13)	Ref	21 (12)	Ref
	Medium	26 (16)	1.65 (0.76-3.60)	21 (11)	1.11 (0.44-2.80)
	High	25 (15)	1.59 (0.69-3.66)	21 (12)	1.89 (0.73-4.92)
aTreg/rTreg (ratio)	Low	26 (14)	Ref	21 (11)	Ref
	Medium	25 (16)	1.06 (0.46-2.43)	21 (12)	0.89 (0.34-2.30)
	High	25 (14)	0.56 (0.24-1.31)	21 (12)	0.40 (0.15-1.06)
			**CSF**		
CD3+	Low	27 (15)	Ref	21 (11)	Ref
	Medium	27 (17)	**2.21 (1.01-4.82)**	21 (14)	**3.22 (1.21-8.57)**
	High	26 (14)	1.24 (0.54-2.82)	21 (10)	1.36 (0.50-3.65)
CD4+	Low	27 (14)	Ref	21 (12)	Ref
	Medium	27 (16)	1.79 (0.82-3.93)	21 (11)	1.57 (0.64-3.83)
	High	26 (16)	**3.04 (1.24-7.46)**	21 (12)	**3.30 (1.08-10.1)**
CD8+	Low	23 (10)	Ref	21 (09)	Ref
	Medium	22 (16)	1.22 (0.47-3.15)	21 (14)	1.32 (0.48-3.61)
	High	22 (11)	0.78 (0.30-2.06)	21 (12)	1.06 (0.39-2.89)
Teff	Low	27 (14)	Ref	21 (12)	Ref
	Medium	26 (15)	1.68 (0.75-3.77)	21 (11)	1.88 (0.76-4.67)
	High	26 (17)	**3.18 (1.33-7.64)**	21 (12)	2.06 (0.76-5.58)
Treg	Low	27 (17)	Ref	21 (14)	Ref
	Medium	26 (13)	0.96 (0.44-2.08)	21 (08)	0.72 (0.29-1.81)
	High	26 (16)	0.87 (0.41-1.86)	21 (13)	0.93 (0.40-2.18)

Supplementary Table 6. Correlations of T cell subsets between blood and cerebrospinal fluid using Spearman's correlation. Number of observations, correlation coefficient and the significance levels are stated for each T cell subtype.

Cell population out of live cells (%)	**No. of patients**	**Spearman's rho**	**P-value**
$CD3^+$	85	0.33	0.0018
$CD4^+$	85	0.31	0.0041
$CD8^+$	71	0.31	0.0092
$CD4^+/CD8^+$ (ratio)	72	0.20	0.0876
T_{eff}	83	0.30	0.0062
Treg	83	0.27	0.0131

Supplementary Methods

5' scRNA-seq with TCR V(D)J: library preparation and sequencing

CSF samples were collected via lumbar puncture and processed within 2 hours after collection. The samples were centrifuged at 400 g for 10 min at 4 °C and resuspended in 50 µl of MACS buffer (1X PBS - calcium and magnesium free - containing 0.04% BSA (400 µg/ml)). 5 µl of the single-cell suspension were then taken out for manual cell counting (Fuchs-Rosenthal chamber). Samples with visible blood contamination or >200 RBCs/µl were excluded.

Single-cell suspensions were then loaded onto Chromium Next GEM Chip G and GEMs were generated after running on the Chromium Single Cell Controller following the instructions from Chromium Next GEM Single Cell V(D)J Reagent Kits v1.1. cDNA was amplified and 5P libraries as well as TCR-enriched libraries were constructed according to manufacturer's instructions. 5P and TCR libraries were then pooled together for sequencing, which was carried out on the NovaSeq sequencer aiming at approximately 50,000 reads per cell. Demultiplexing, quality control and transcript counting were performed by Cell Ranger Pipeline (v3.1.0; 10x Genomics), using the default parameters. The reference for alignment is GRCh38 genome. The sample processing described above, and cell ranger computations were performed at Eukaryotic Single Cell Genomics (ESCG), ScilifeLab, Karolinska Institutet.

scRNA-seq data processing, integration, and dimension reduction

scRNA-seq allows to define the transcriptomic profiles of the CSF cells to determine if they exhibited any differences reflecting phenotypic variations. Post-filtering with Cell Ranger to remove background noise and to retain only cells with valid cellular barcodes, filtered gene-cell count matrix for each sequenced CSF sample was generated and furthered processed with Seurat v4.0.4[1], using R v4.1.0. Secondary filtering was carried out with Seurat to remove low-quality cells with over 5% mitochondrial gene expressed or cells with fewer than 200 genes or more than 20,000 genes. A total of 28,867 cells, including 15,195 cells from ALS patients and 13,672 cells from controls, were retained for downstream analyses. Log-normalization was carried out using Seurat using *NormalizeData* function on the gene count data of the remaining cells. Highly variable genes (N=2,000) were selected using *FindVariableFeatures* function in Seurat and used for scaling the data and projection of data into lower-dimensional space using Principle Component Analysis (PCA). Integration was done using the harmony algorithm[2], by

utilising PCA embeddings of the data, and returned a set of corrected embeddings, taking batch information into consideration. Dimension reduction using t-distributed Stochastic Neighbor Embedding (tSNE) was performed with *RunTSNE* function in Seurat, using the first 30 harmony-corrected embeddings.

Differential gene expression (DE) analysis and abundance comparison

In this analysis, we focused on T cells. For each annotated T cell type, DE analyses were performed using *FindAllMarkers* function (default parameters) for comparisons between 1) ALS patients and 2) controls. Wilcoxon rank sum test was conducted and p-values were adjusted using Bonferroni correction and only DE genes with p-adjusted value <0.05 and average log2-fold change >0 were considered. Two-proportions Z-test was conducted separately for comparisons 1) and 2) for each annotated cell type (only cell types with more than 5 cells in each group were considered) and log10-fold change was computed using the fold change of the mean proportion of the cell type in group 1 to the mean proportion of the same cell type in group 2.

TCR repertoire clonal analysis

scRepertoire v1.3.2 was used to conduct the clonal analysis with scRNA-seq data[3]. Clonal space homeostasis was computed for each detectable T cell, based on their VDJC genes and the CDR3 nucleotide sequence, using *clonalHomeostasis* function. tSNE was recomputed using only cells with captured TCR information and homeostasis status was projected onto the new tSNE visualization with contours to show homeostasis densities using function *clonalOverlay* (parameters: freq.cutpoint=30, bins=20). Cells with clonal frequency >30 were indicated using contours. Cell proportion of each homeostasis level for each cell type was computed using *occupiedscRepertoire* function and statistical analysis was conducted to compare the cell numbers in cell types between ALS patients and controls using Chi-square test. Chi-square test was also used to determine whether differences were significant between expanded and nonexpanded cells in ALS.

Supplementary References

1. Hao, Y. *et al.* Integrated analysis of multimodal single-cell data. *Cell* **184**, 3573-3587.e29 (2021).
2. Korsunsky, I. *et al.* Fast, sensitive and accurate integration of single-cell data with Harmony. *Nat Methods* **16**, 1289–1296 (2019).
3. Borcherding, N. & Bormann, N. L. scRepertoire: An R-based toolkit for single-cell immune receptor analysis. (2020) doi:10.12688/f1000research.22139.1.
