# Supplementary material for: T cell responses at diagnosis of amyotrophic lateral sclerosis predict disease progression
Source: Nat Commun. 2022 Nov 8;13:6733. doi: 10.1038/s41467-022-34526-9 (PMC9643478; doi:10.1038/s41467-022-34526-9)
Supplement: Supplementary file 3 — Reporting Summary [file 41467_2022_34526_MOESM3_ESM.pdf]

nature portfolio

Corresponding author(s): Fang Fang
John Andersson

Last updated by author(s): Jul 4, 2022

Reporting Summary

Nature Portfolio wishes to improve the reproducibility of the work that we publish. This form provides structure for consistency and transparency in reporting. For further information on Nature Portfolio policies, see our Editorial Policies and the Editorial Policy Checklist.

Statistics

For all statistical analyses, confirm that the following items are present in the figure legend, table legend, main text, or Methods section.

n/a	Confirmed	
☐	☒	The exact sample size (n) for each experimental group/condition, given as a discrete number and unit of measurement
☒	☐	A statement on whether measurements were taken from distinct samples or whether the same sample was measured repeatedly
☐	☒	The statistical test(s) used AND whether they are one- or two-sided<br>*Only common tests should be described solely by name; describe more complex techniques in the Methods section.*
☐	☒	A description of all covariates tested
☐	☒	A description of any assumptions or corrections, such as tests of normality and adjustment for multiple comparisons
☐	☒	A full description of the statistical parameters including central tendency (e.g. means) or other basic estimates (e.g. regression coefficient) AND variation (e.g. standard deviation) or associated estimates of uncertainty (e.g. confidence intervals)
☐	☒	For null hypothesis testing, the test statistic (e.g. F, t, r) with confidence intervals, effect sizes, degrees of freedom and P value noted<br>*Give P values as exact values whenever suitable.*
☒	☐	For Bayesian analysis, information on the choice of priors and Markov chain Monte Carlo settings
☒	☐	For hierarchical and complex designs, identification of the appropriate level for tests and full reporting of outcomes
☐	☒	Estimates of effect sizes (e.g. Cohen's d, Pearson's r), indicating how they were calculated

Our web collection on statistics for biologists contains articles on many of the points above.

Software and code

Policy information about availability of computer code

Data collection	No software was used.

Data analysis

Flow cytometry data:
FlowJo 10 (BD Biosciences) was used for quantifying the cell proportions.
We first used Cox model to calculate the hazard ratios (HRs) and their 95% confidence intervals (CIs) of death (alternatively use of invasive ventilation) in relation to the frequencies of T cell subsets, per standard deviation (SD) increase, with time since diagnosis as the underlying time scale. Afterward, we used Spearman's correlation to assess the correlation of T cell subsets with disease progression rate at the time of diagnosis. We also performed an exploratory factor analysis using mifa R package v. 0.2.0 to understand the synergistic effect of different immune markers. Finally, to assess whether the studied T cell subsets could help to stratify unique patient groups, we performed a cluster analysis by using k-means clustering.
Single cell RNA sequencing data:
For single cell RNA sequencing demultiplexing, quality control and transcript counting were performed by Cell Ranger 19 Pipeline (v3.1.0; 10x Genomics), using the default parameters. The reference for alignment is 20 GRCh38 genome. Post-filtering with Cell Ranger to remove background noise and to retain only cells with valid cellular barcodes, filtered gene-cell count matrix for each sequenced CSF sample was generated and furthered processed with Seurat v4.0.4, using R v4.1.0. Log-normalization was carried out using Seurat using NormalizeData function on the gene count data. Highly variable genes (N=2,000) were selected using FindVariableFeatures function in Seurat and used for scaling the data and projection of data into lower-dimensional space using Principle Component Analysis (PCA). Integration was done using the harmony algorithm v. 1.0, by utilising PCA embeddings of the data, and returned a set of corrected embeddings, taking batch information into consideration. Dimension reduction using t-distributed Stochastic Neighbor Embedding (tSNE) was performed with RunTSNE function in Seurat, using the first 30 harmony-corrected embeddings. In this analysis, we focused on T cells. For each annotated T cell type, DE analyses were performed using FindAllMarkers function (default parameters) for comparisons between ALS patients and controls. scRepertoire v1.3.2 was used to conduct the clonal analysis with scRNA-seq data. The code for this section of the analysis can be found at: https://github.com/eudoraleer/als_scrnaseq. (DOI:10.5281/zenodo.6759696)

For manuscripts utilizing custom algorithms or software that are central to the research but not yet described in published literature, software must be made available to editors and reviewers. We strongly encourage code deposition in a community repository (e.g. GitHub). See the Nature Portfolio guidelines for submitting code & software for further information.

Data

Policy information about availability of data

All manuscripts must include a data availability statement. This statement should provide the following information, where applicable:

- Accession codes, unique identifiers, or web links for publicly available datasets
- A description of any restrictions on data availability
- For clinical datasets or third party data, please ensure that the statement adheres to our policy

Due to ethical and legal reasons, the flow cytometric and clinical data of the ALS patients are not publicly available. Such data might however be shared on reasonable request to the corresponding authors. The RNAseq data and a description of the data have been deposited at the Swedish National Data Service (https://doi.org/10.48723/xjvx-2v24).

Field-specific reporting

Please select the one below that is the best fit for your research. If you are not sure, read the appropriate sections before making your selection.

☒ Life sciences ☐ Behavioural & social sciences ☐ Ecological, evolutionary & environmental sciences

For a reference copy of the document with all sections, see nature.com/documents/nr-reporting-summary-flat.pdf

Life sciences study design

All studies must disclose on these points even when the disclosure is negative.

Sample size

The study was initiated in March 2016 and patients samples were collected until March 2020. The sample size was not predefined, we included as many patients as practically possible until COVID became increasingly prevalent in the study area. We believe extending the time line may cause more harm than good as viral infections have a stronger impact on immunity than ALS.

Data exclusions

Data was excluded if there were technical problems with sample preparation, i.e. loss of sample, or if the flow cytometry staining failed.

Replication

Replicating data from a patient study like this, that requires analysis of fresh samples from both blood and CSF, is not feasible due to the rarity of the disease, the invasiveness of the required samples and the high morality rate. Instead we have designed a very robust flow cytometry panel that was extensively tested before the start of the study.

Randomization

The comparisons for the flow cytometry are made within a single group of patients and in the single cell RNA sequencing we compare different diseases. In no case we were comparing experimental groups that requires randomization upon inclusion.

Blinding

In the flow cytometry analysis all samples came from ALS patients and gating was performed without knowledge of disease progression rate and disease outcome. In the single cell RNA sequencing the annotation was performed on all sequenced cells in a single tSNE plot without knowledge of which cells belonged to what sample.

Reporting for specific materials, systems and methods

We require information from authors about some types of materials, experimental systems and methods used in many studies. Here, indicate whether each material, system or method listed is relevant to your study. If you are not sure if a list item applies to your research, read the appropriate section before selecting a response.

Materials & experimental systems

n/a	Involved in the study
☐	☒ Antibodies
☒	☐ Eukaryotic cell lines
☒	☐ Palaeontology and archaeology
☒	☐ Animals and other organisms
☐	☒ Human research participants
☒	☐ Clinical data
☒	☐ Dual use research of concern

Methods

n/a	Involved in the study
☒	☐ ChIP-seq
☐	☒ Flow cytometry
☒	☐ MRI-based neuroimaging

Antibodies

Antibodies used	Antibodies (Fluorochrome, Clone, Provider, Reference, Amount used per 1 million cells): CD3 (AF488, HIT3a, BioLegend, 300320, 2 uL), CD4 (BV786, SK3, BD Biosciences, 563877, 2 uL), CD8 (BV605, SK1, BD Biosciences, 564116, 3 uL), CD127 (PE-Cy7, eBioRDR5, ThermoFisher, 25-1278-42, 0.4 uL), CD25 (PE, 3G10, ThermoFisher, MHCD2504, 1 uL), CD45RA (APC-H7, HI100, BioLegend, 304150, 0.4 uL), FoxP3 (PE-CF594, 236A/E7, BD Biosciences, 653955, 5 uL) and Ki67 (BV421, B56, BD Biosciences, 562899, 5 uL).
Validation	The antibodies used herein are commercially available and extensively used throughout the research community. Additional information of the clones can be found in:<br><br>HIT3a is characterized in: 1) Schlossman S, et al. Eds. 1995. Leucocyte Typing V. Oxford University Press. New York. 2) Knapp W. 1989. Leucocyte Typing IV. Oxford University Press New York. 3) Barclay N, et al. 1997. The Leucocyte Antigen Facts Book. Academic Press Inc. San Diego.<br><br>SK1 and SK3 are described in Bernard A, Boumsell L, Hill C. Joint report of the first international workshop on human leucocyte differentiation antigens by the investigators of the participating laboratories. In: Bernard A, Boumsell L, Dausset J, Milstein C, Schlossman SF, eds. Leucocyte Typing. New York, NY: Springer-Verlag; 1984:9- 108.<br><br>3G10 is characterized in: Goldstein AM, Marcon L, Cullen BR and Nelson DL.1988 J.Immunol. Methods 107:103<br><br>eBioRDR5 was used in Hartigan-O'Connor et al. Journal of Immunological Methods, 2007, 319:41-52.<br><br>HI100, BioLegend is discussed in Knapp W, et al. 1989. Leucocyte Typing IV. Oxford University Press. New York<br><br>236A/E7 is described in Roncador et al. Eur J Immunol, 35:1681-91, 2005.<br><br>Flow cytometry for Ki67 is described in Cell cycle dependent expression and stability of the nuclear protein detected by Ki-67 antibody in HL-60 cells. Cell proliferation, 1992, 25:31-40.

Human research participants

Policy information about studies involving human research participants

Population characteristics	The population characteristics are available in Table 1.
Recruitment	All ALS patients that were diagnosed between March 2016 and March 2020 were asked to participate in the study. The participation was voluntarily and upon signing an informed consent. Since the collection of blood and CSF was part of the routine diagnostic workup and the patients are generally not too ill at the time of diagnosis, we tend to have a high participation rate (above 90%). Importantly, to make sure there are minimal sources of bias in our selection criteria, the characteristics of this patient cohort have been compared to all ALS patients diagnosed in this area during the same time period and the data are shown in Table 1.
Ethics oversight	The study was approved by the Ethical Review Board in Stockholm, Sweden (DNRs 2014/1815-31/4 and 2018-1065/31)

Note that full information on the approval of the study protocol must also be provided in the manuscript.

Flow Cytometry

Plots

Confirm that:

☒ The axis labels state the marker and fluorochrome used (e.g. CD4-FITC).

☒ The axis scales are clearly visible. Include numbers along axes only for bottom left plot of group (a 'group' is an analysis of identical markers).

☒ All plots are contour plots with outliers or pseudocolor plots.

☒ A numerical value for number of cells or percentage (with statistics) is provided.

Methodology

Sample preparation	Listed in methods:<br><br>Blood and CSF samples were collected at the time of diagnosis or shortly thereafter (within three months after diagnosis) for all study participants. These consisted of a 3mL blood sample collected in a sodium heparin tube (BD) and a 16mL sample collected in two 10mL plastic tubes (Sarstedt) by lumbar puncture. Blood was kept at room temperature, while CSF was directly centrifuged (400g, 10min) with the isolated cells kept at 4°C. Both specimens were processed fresh without intervening freezing. The average time between the sampling and the start of the experimental analysis was around two hours. Peripheral blood mononuclear cells (PBMCs) were separated using a Ficoll (GE Healthcare) density gradient centrifugation according to the manufacturer’s protocol. PBMCs were washed with PBS and a total of 1 million cells were collected for further analysis. All CSF cells were washed with PBS before further analysis.
Instrument	Data were acquired on BD LSRFortessa (BD Biosciences).
Software	Data was analyzed using FlowJo 10 (BD Biosciences).
Cell population abundance	Not currently listed.
Gating strategy	The gating strategy is provided in supplementary figure 1.

☒ Tick this box to confirm that a figure exemplifying the gating strategy is provided in the Supplementary Information.
